# FINE, a novel laboratory-based frailty index for elderly patients: a retrospective descriptive study

**DOI:** 10.1590/1516-3180.2025.3337.13022026

**Published:** 2026-05-01

**Authors:** Yasin Altun, Halime Dilber Balci, Nilay Çom Aybal

**Affiliations:** I Asst. Professor, Department of Family Medicine, Faculty of Medicine, Niğde Ömer Halisdemir Üniversitesi, Niğde, Türkiye.; II Physician, Department of Family Medicine, Niğde Ömer Halisdemir Üniversitesi Research and Training Hospital, Niğde, Türkiye.; III Department of Family Medicine, Faculty of Medicine, Yalova Üniversitesi, Yalova, Türkiye.

**Keywords:** Frailty, Biomarkers, Elderly, Geriatric assessment, FINE score, Clinical frailty scale, Primary care

## Abstract

**BACKGROUND:**

Frailty in older adults is a multifactorial geriatric syndrome associated with inflammation, malnutrition, and hematological decline. Objective and easily applicable laboratory-based indices may complement clinical frailty assessment by providing rapid and low-cost screening tools, particularly in primary care and resource-limited settings.

**OBJECTIVES:**

To develop a simple laboratory-based frailty screening index (FINE, Frailty Index for the Elderly) using C-reactive protein (CRP), albumin, hemoglobin, and sex, and to evaluate its association with the Clinical Frailty Scale (CFS) in older adults.

**DESIGN AND SETTING:**

A retrospective descriptive study conducted using electronic health records of individuals aged 80 years and older.

**METHODS:**

Data from 322 older adults were analyzed. Their FINE scores were calculated by assigning 0 or 1 point to CRP, albumin, hemoglobin, and sex based on clinically accepted reference thresholds, yielding a total score ranging from 0 to 4. Frailty was assessed using pre-recorded CFS scores. Associations between FINE scores, CFS, and individual biomarkers were examined. The screening performance was evaluated using receiver operating characteristic (ROC) curve analysis.

**RESULTS:**

The mean age of participants was 84.9 ± 4.0 years, and 55.6% were female. The prevalence rate of frailty was 46.6%. FINE scores exhibited a positive correlation with CFS and CRP levels, and a negative correlation with albumin and hemoglobin levels (p < 0.005). ROC analysis demonstrated a statistically significant but moderate discriminatory ability for frailty (area under the curve = 0.642; 95% confidence interval: 0.5820.703). At a cut-off value of ≥ 0.5, FINE scores demonstrated high sensitivity (89.3%) but low specificity (22.1%).

**CONCLUSION:**

The FINE score is a simple, rapid, and low-cost laboratory-based frailty screening tool that is significantly associated with clinical frailty and key biological processes underlying frailty. Although low specificity limits its use as a diagnostic instrument, it may serve as a practical first-step screening approach in primary care and resource-limited settings. Further multicenter prospective studies are required to validate these findings.

## INTRODUCTION

 Frailty is a multidimensional geriatric syndrome characterized by progressive decline in physiological reserves and increased vulnerability to internal and external stressors. It is closely associated with adverse health outcomes including falls, hospitalization, functional dependency, institutionalization, and mortality. The biological mechanisms underlying frailty include chronic low-grade inflammation, immune system dysregulation, malnutrition, and hematological impairment.^1,2^

 The Clinical Frailty Scale (CFS) is one of the most widely used tools for assessing frailty in clinical practice. It provides a rapid global assessment based on functional status and clinical judgment.^3^ Although such clinical scales are easy to apply, they may have limitations, such as variability due to cognitive bias of the evaluator. Given the rapid increase in the older population, more objective, quantitative, and reproducible methods are needed to determine frailty.^4^

 Laboratory biomarkers have recently gained attention as potential indicators of frailty. Elevated levels of inflammatory markers such as C-reactive protein (CRP), reduced serum albumin levels, and decreased hemoglobin concentrations have consistently been associated with frailty and adverse geriatric outcomes.^5-7^ These biomarkers reflect key pathophysiological domains of frailty, including systemic inflammation, nutritional status, and hematological reserve, and are routinely measured in clinical practice. 

 In addition, frailty is more common in women than in men and frailty scores are higher in women; this difference has been attributed to biological and socioeconomic factors such as lifespan, physical limitations, hormonal changes, and levels of social support.^8,9^ While sex itself is not a biological marker, it may modify the expression and clinical manifestations of frailty. 

 Several laboratory-based frailty indices have been proposed. However, many of these models rely on a large number of laboratory variables, which may limit their feasibility and routine use in everyday clinical settings.^10^ Therefore, a simple, laboratory-based frailty screening tool that prioritizes accessibility, rapid calculation, and applicability in primary care and resource-limited environments is highly needed. 

 In this context, we developed the Frailty Index for the Elderly (FINE), a concise laboratory-based score derived from three routinely available biomarkers (CRP, albumin, and hemoglobin), and a sex component. Rather than replacing comprehensive clinical frailty assessments, the FINE score is intended to be a screening-oriented tool to help identify older individuals who may benefit from further geriatric evaluation. In this study, we sought to examine the association between the FINE score and clinical frailty as assessed using the CFS, evaluate its screening performance using receiver operating characteristic (ROC) analysis, and explore its potential utility in adults aged 80 years and above. 

## OBJECTIVE

 The primary objective of this study was to develop a simple, laboratory-based frailty index (FINE ) using CRP, albumin, hemoglobin, and sex and to evaluate its association with CFS in older adults. 

## METHODS

### Study design and population

 This retrospective, single-center study included individuals aged 80 years and older who were evaluated at the Healthy Aging Unit and were followed for at least one year. Inclusion criteria were as follows: ≥ 80 years old, at least one complete blood count and biochemical test performed, and the presence of CFS data in their medical records. Participants’ clinical data and laboratory parameters were reviewed, and their relationships with frailty were evaluated. 

 Demographic characteristics (age and sex), comorbidity status, and clinical observations of the participants were obtained from the patient records system. The laboratory parameters evaluated as biomarkers were CRP (mg/L), albumin (g/dL), and hemoglobin (g/dL). 

 All biochemical data were obtained from initial blood samples collected at the time of admission from the study participants. Laboratory data from a single time point were analyzed for each individual. Measurements were performed in the hospital laboratory using automated analyzers. 

 This was a retrospective, descriptive file review study. The study was conducted in accordance with the Declaration of Helsinki and approved by the Niğde Ömer Halisdemir Üniversitesi Research Ethics Review Committee (Approval No 2025/22). Patient identity information was kept confidential and used only for scientific purposes. 

### Data collection and definitions

 FINE scores were established using three biomarkers (CRP, albumin, and hemoglobin) and sex. To enhance clinical interpretability and external applicability, cut-off values for each biomarker were determined based on commonly accepted clinical reference thresholds routinely used in geriatric practice, rather than being statistically derived from the study population. Values below or above threshold values were assigned 0 or 1 point, respectively, and a total FINE score ranging from 0 to 4 was calculated for each participant (**Table 1**). Higher scores indicated increased biological frailty. Sex was included as a component of the score based on a well-established epidemiological evidence indicating wellestablished epidemiological evidence women, reflecting population-level biological vulnerability. 

**Table 1:** FINE score = CRP + albumin+ hemoglobin + sex

**Parameter**	**Value**	**Point**
CRP	≤ 5 mg/L	0
	> 5 mg/L	1
Albumin	≥ 35 g/L	0
	< 35 g/L	1
Hemoglobin	≥ 12 g/dL (female)	0
	≥ 13 g/dL(male)	
	Lower than	1
Sex	Male	0
	Female	1

FINE, Frailty Index for the Elderly; CRP, C-reactive protein.

### Clinical frailty assessment

 The level of clinical frailty was assessed using CFS scores, which were previously entered into the patient records system. The CFS is a widely used and highly valid measure of frailty that rates individuals on a scale from 1 (very fit) to 9 (terminally ill).^11,12^ Frailty was operationally defined as a CFS score ≥ 5, corresponding to at least mild frailty, while scores of 1–4 were considered non-frail. 

### Statistical analysis

 Data analysis was performed using IBM SPSS Statistics for Windows, version 25.0 (Armonk, New York). The normality of continuous data was assessed using the Kolmogorov–Smirnov test. Normally distributed continuous data are presented as means ± standard deviation, non-normally distributed data as median (minimum-maximum), and categorical variables as frequency and percentage. For comparisons between two groups of continuous data, the Mann–Whitney *U* test was used for independent groups, and the Kruskal–Wallis test was employed for comparisons between three groups. The Games–Howell test was used for post-hoc analysis to determine statistical significance between the groups. As conditions for parametric tests were not met, Spearman’s rank correlation analysis was used to examine the relationships. ROC curve analysis was applied to determine the diagnostic accuracy and to identify the most appropriate cutoff points for the variables. Within the scope of the ROC analysis, sensitivity and specificity were evaluated at different test threshold values. The area under the ROC curve (AUC) was calculated to evaluate the classification success of the model. In all statistical analyses, statistical significance is set at a p-value of < 0.05. Due to the retrospective study design and sample size, formal internal and external validation analyses were not performed. 

## RESULTS

 The average age of the participants was 84.86 ± 4.01 years, and 55.6% were female. The most common chronic diseases were cardiovascular (45.8%), endocrine (16.2%), and gastrointestinal (10.4%) disorders. The average scores of the CFS, Katz Activities of Daily Living Scale (Katz ADL), and Lawton Instrumental Activities of Daily Living (Lawton IADL) Scale are presented in **Table 2**. 

**Table 2:** Clinical and functional assessment score averages

	**Mean + SD**	**Median (min–max)**
Age	84.86 ± 4.01	84(80–102)
CFS score	4.4 ± 1.41	4(1–9)
Katz ADL	4.78 ± 1.48	5(0–6)
Lawton IADL	6 ± 2.35	7(0–9)
CRP(mg/L)	8.87 ± 19.93	2.85(0.1–143.8)
Albumin (g/L)	40.92 ± 4.11	41(22–50)
Hemoglobin (g/dL)	12.74 ± 1.82	12.8(6.9–17.3)
FINE score	1.38 ± 0.93	1(0–4)

FINE, Frailty Index for the Elderly; CFS, Clinical Frailty Scale; ADL, Activities of Daily Living; IADL, Instrumental Activities of Daily Living; CRP, C-reactive protein.

 Of all the participants, 46.6% were assessed as frail. According to Katz ADL scores, 60.6% of participants were partially dependent, whereas according to the Lawton IADL score, this percentage was 95.7%. **Table 3** shows the distribution of frailty status according to the calculated mean FINE scores and determined cut-off values. 

**Table 3:** Distribution of fragility based on participant features and FINE scores

		**N**	**%**
Gender	Female	179	55.6
	Male	143	44.4
Frailty status according to CFS	Terminally ill	1	0.3
	Living with very severe frailty	6	1.9
	Living with severe frailty	8	2.5
	Living with moderate frailty	53	16.5
	Living with mild moderate frailty	82	25.5
	Living with very mild frailty	94	29.2
	Managing well	57	17.7
	Fit	8	2.5
	Very fit	13	4
Presence of frailty based on CFS	Fragile	150	46.6
	Not fragile	172	53.4
Dependency status according to Katz ADL score	Independent	118	36.6
	Moderate impairment	195	60.6
	Very dependent	9	2.8
Dependency status according to Lawton IADL score	Independent	1	0.3
	Moderate impairment	308	95.7
	Very dependent	13	4
Anemic condition	Anemia present	191	59.3
	Anemia absent	131	40.7
Presence of chronic disease	Cardio vascular system disease	232	45.8
	Endocrine system disease	82	16.2
	Neurologic disease	46	9
	Gastro intestinal system disease	53	10.4
	Respiratory system disease	42	8.3
	Rheumatologic disease	17	3.3
	Other diseases	8	1.5
	No chronic disease	26	5.1
FINE score-based fragility	FINE score ≥ 0.5(fragile)	268	83,2
	FINE score < 0.5 (not fragile)	54	16,8

FINE, Frailty Index for the Elderly; CFS, Clinical Frailty Scale; ADL, Activities of Daily Living; IADL, Instrumental Activities of Daily Living.

 A significant positive correlation was identified between the participants’ CFS scores and both CRP and FINE scores, whereas albumin and hemoglobin levels were negatively correlated (p < 0.005). Katz ADL scores were negatively correlated with CRP and FINE scores and positively correlated with albumin and hemoglobin values (p < 0.005). Similarly, Lawton IADL scores were negatively correlated with CRP and FINE scores and positively correlated with albumin and hemoglobin levels (p < 0.005) (**Table 4**). 

**Table 4:** Correlations between clinical and functional scales and laboratory parameters

		**Age**	**CFS**	**Katz ADL**	**Lawton IADL**
CRP (mg/L)	r	−0.029	0.155	−0.136	−0.134
	p^*^	0.606	0.005	0.015	0.016
Albumin (g/L)	r	−0.19	−0.327	0.238	0.342
	p^*^	0.001	< 0.001	< 0.001	< 0.001
Hemoglobin (g/dL)	r	−0.074	−0.164	0.199	0.174
	p^*^	0.185	0.003	< 0.001	0.002
FINE	r	0.068	0.314	−0.319	−0.26
	p^*^	0.225	< 0.001	< 0.001	< 0.001

* Spearman’s correlation test;

CRP, C-reactive protein.

 Frail participants had higher CRP levels and lower albumin and hemoglobin levels. A significant difference was found between the participants’ dependency status, as measured by their Katz ADL scores, and their albumin and hemoglobin levels. The post-hoc analysis revealed that the albumin and hemoglobin levels of fully independent participants were higher than those of participants with moderate impairment (**Table 5**). 

**Table 5:** Comparison of laboratory values according to frailty and dependency status

		**CRP (mg/L)**		**Albumin (g/L)**		**Hemoglobin (g/dL)**	
		**Median (min-max)**	**p**	**Median (min-max)**	**p**	**Median (min-max)**	**p**
Gender								
	*Female*	2.70 (0.20–143.8)	0.763^a^	42 (26–48)	0.123^a^	12.4 (6.9–16.4)	**< 0.001^a^**
	*Male*	3 (0.1–129.3)		41 (22–50)		13.3(9.1–17.3)	
Presence of frailty based on CFS								
	*Fragile*	3.35 (0.3–143.8)	**0.033^a^**	40 (22–48)	**< 0.001^a^**	12.5 (6.9–17)	**0.006^a^**
	*Not fragile*	2.3 (0.1–132.3)		42 (26–50)		13 (8.4–17.3)	
Dependency status according to Katz ADL								
	*Independent*	2.3 (0.1–97.5)	0.164^b^	42 (26–50)	**0.036^c^ (a–b)**	13.08 (8.4–17.3)	**0.030^c^ (a–b)**
	*Moderate impairment*	3.1 (0.2–143.8)		41 (22–48)		12.6 (6.9–17)	
	*Very dependent*	3.2 (1.2–14.2)		38 (28–46)		13.1 (9.7–14)	
Dependency status according to Lawton IADL								
	*Independent and moderate impairment*	2.8 (0.1–143.8)	0.678^a^	42 (22–50)	0.081^a^	12.8 (6.9–17.3)	0.45^a^
	*Very dependent*	3.9 (0.6–14.2)		40 (28–46)		13.1 (9.7–14)	

a Mann–Whitney U test;

b Kruscal–Wallis test;

c Games–Howel Test;

FINE, Frailty Index for the Elderly; CFS, Clinical Frailty Scale; ADL, Activities of Daily Living; IADL, Instrumental Activities of Daily Living; CRP, C-reactive protein.

**Figure 1:**
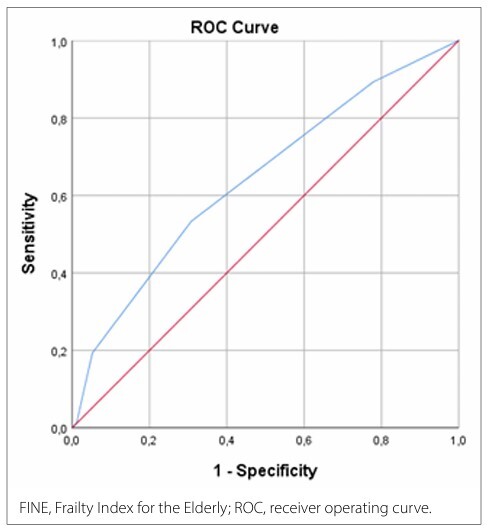
ROC curve analyses of FINE.

 As shown in **Table 6**, the FINE score was statistically significant in distinguishing frailty (p < 0.001). The AUC for diagnosing the presence of frailty with the FINE score was 0.642 (95% CI, 0.582−0.703). (**Figure 1**). At a cutoff value of ≥ 0.50, the sensitivity of FINE for predicting the presence of frailty was 89.3% and its specificity was 22.1%. 

**Table 6:** Discriminative power of FINE scores in frailty diagnosis (ROC analysis)

	**AUC**	**95% CI**	**Cut-off**	**Sensitivity (%)**	**Specificity (%)**	**p**
FINE	0.642	0.582–0.703	≥ 0.5	89.3	22.1	< 0.001

## DISCUSSION

 The FINE score developed in this study represents a practical and feasible laboratory-based approach for frailty screening in older adults. Based on only three routinely available biomarkers and sex, the FINE score demonstrated a significant association with clinical frailty, as assessed by the CFS in individuals aged ≥ 80 years. The AUC values obtained in the ROC analysis demonstrated that the FINE score provided meaningful accuracy in distinguishing biological frailty levels. These findings support those of previous studies, suggesting that frailty screening in older adults can be performed using simple biomarkers.^12,13^

 Frailty is strongly linked to biological processes such as systemic inflammation, nutritional deterioration, and hematological decline.^14^ The three key biomarkers used in our study (CRP, albumin, and hemoglobin) represent the pathophysiological components of frailty. Numerous studies have shown that CRP levels are significantly elevated in frail individuals, indicating that systemic inflammation contributes to increased physiological frailty in older adults.^5,15^ These findings underscore the pathophysiological significance of including CRP in the FINE scoring system. Similarly, low albumin levels are directly associated with both frailty and mortality and are considered an indicator of overall health in older individuals.^16,17^ Hemoglobin levels are associated with both nutritional status and physical capacity in older adults. Reduced oxygen-carrying capacity in anemic individuals may lead to functional loss and an increased risk of falls.^18^ According to the Toulouse cohort study, a 1 g/dL increase in hemoglobin was associated with a 14% reduction in the risk of frailty (OR = 0.86; p < 0.005).^19^ These results support the association between low hemoglobin levels, loss of physiological reserve, and functional impairment. In this context, the ability of the FINE score to combine these three parameters into a simplified structure reflecting both the biological frailty burden and systemic physiological reserve is an important contribution. 

 Various laboratory-based frailty indices have been proposed. For example, models such as FI-LAB use dozens of parameters, which limit their applicability in daily practice.^20,21^ In a study of 26,554 patients in France, the bFRAil score was used and was based on CRP, hemoglobin, albumin, vitamin D, age, and sex. The bFRAil score demonstrated a strong diagnostic performance, with an AUC of 0.78.^22^ However, calculating this score is complex and time-consuming. Compared with more complex laboratory frailty indices, FINE prioritizes feasibility and accessibility. This simplicity may be particularly advantageous in primary care, home healthcare, and settings with limited resources. 

 The discriminatory performance of FINE was moderate (AUC, 0.642), suggesting that it should be viewed as a screening-oriented tool rather than as a diagnostic instrument. Its high sensitivity (89.3%) supports its potential role in identifying individuals who may benefit from comprehensive geriatric assessment. These features make FINE a fast, low-cost, and widely accessible approach to assessing frailty. It enables frailty screening using only routine laboratory data without the need for complex physical tests or time-consuming functional assessments. The FINE score stands out as a practical and applicable tool, particularly in primary care services, home healthcare, and clinical settings with limited resources. Through the integrated use of biological markers, age-specific pathophysiological processes such as systemic inflammation, nutritional status, and hematological reserve can be objectively assessed. 

 This study had certain limitations. The retrospective, singlecenter design limits generalizability, and potential confounders such as comorbidity burden and functional dependency were not analytically adjusted for. In addition, no external validation was performed. Future multicenter prospective studies are needed to validate the predictive value of FINE and assess its association with longitudinal clinical outcomes. 

## CONCLUSION

 FINE is a simple, low-cost, laboratory-based frailty screening tool that demonstrates a significant association with clinical frailty in older adults. Although not intended as a diagnostic instrument, it may serve as a practical first-step screening approach to identify individuals at increased risk of frailty. Future multicenter prospective studies are warranted to validate its predictive and prognostic value for clinical outcomes. 

## Data Availability

Data supporting the findings of this study are available from the corresponding author, Yasin Altun, upon request.
